# Is chronic urticaria more than skin deep?

**DOI:** 10.1186/s13601-019-0287-2

**Published:** 2019-09-25

**Authors:** Emek Kocatürk, Clive Grattan

**Affiliations:** 1 Department of Dermatology, Koç University, School of Medicine, Istanbul, Turkey; 2 St John’s Institute of Dermatology, Guy’s Hospital, London, UK

**Keywords:** Urticaria, Organ involvement, Systemic findings, Cardiac, Gastrointestinal, Respiratory, Brain

## Abstract

Chronic urticaria is a disease characterized by the appearance of weals, angioedema or both longer than 6 weeks. Degranulation of cutaneous or submucosal mast cells leads to release of mediators including histamine resulting in redness, swelling and itch. Because mast cells are widely distributed throughout the body, the question is why they are not activated systemically or does systemic activation occur without overt end organ dysfunction? We have conducted an exploratory literature search for reports that have evidence of organ-specific dysfunction in chronic urticaria that might justify prospective observational studies. This search revealed some evidence of systemic effects of chronic urticaria in cardiac, respiratory, gastrointestinal, central nervous and musculo-skeletal systems. The relevance of these findings needs to be further determined. However, they justify prospective studies in larger numbers of patients and at different stages of disease activity.

## Background

Chronic urticaria (CU) is a disease characterized by the appearance of weals, angioedema or both in which mast cells have a central role [1]. Degranulation of cutaneous or submucosal mast cells results in release of pre-formed and newly synthesised mediators including histamine and cysteinyl leukotrienes LTC4, D4 and E4 resulting in redness, swelling and itch [2]. Because mast cells are widely distributed throughout the body, the question is why they are not activated systemically or does systemic activation occur without overt end organ dysfunction? A possible explanation could be relative restriction of C5a receptor expression to cutaneous mast cells, with preferential activation after C5a generation [3]. Degranulation of human foreskin mast cells required binding of the FcεRI and activation of the classical complement cascade in vitro [4]. Total, but not mature (β), tryptase in the sera of CU patients was higher than healthy and atopic controls, particularly in a subgroup with autoimmune urticaria, and symptomatic rather than asymptomatic patients suggesting an increased systemic mast cell burden [5]. Increased levels of plasma histamine have been reported in CSU patients [6]. Increased plasma histamine could lead to concentration-related histamine-mediated symptoms such as increasing gastric acid secretion and heart rate, tachycardia, headache, flush, urticaria, pruritus, decreased arterial pressure, bronchospasm, and cardiac arrest [7].

Some chronic spontaneous urticaria (CSU) patients have gastrointestinal symptoms, flushing, joint pain or swelling, palpitations, headache/fatigue and wheezing during active urticarial flares suggesting systemic as well as cutaneous mast cell activation [8]. In an epidemiological study, extracutaneous symptoms were found in over a third of cases (39.4%) [9].

We have conducted an exploratory literature search using PubMed and Google Scholar before May 2019 for reports that have evidence of organ-specific dysfunction in CU (spontaneous and inducible) that might justify prospective observational studies. Because the terminology of CU has historically been inconsistent we have included the specific terminology used in each report, accepting that chronic idiopathic urticaria (CIU), chronic spontaneous urticaria (CSU) and even CU itself relate to the same patient population unless specific subtypes of inducible urticaria have been specified. We performed a literature search strategy for evidence including every organ system of the body (Table 1). Reports relating to acute urticaria, urticarial vasculitis, autoinflammatory syndromes, mastocytosis and bradykininergic angioedema were excluded. Many systemic reactions related to inducible urticarias for example laryngeal oedema due to cold urticaria were also excluded. Identified reports that are relevant are summarised in Table 2.

**Table 1:** Studies investigating systems involved in chronic urticaria

Keywords	Search in Pubmed, number of publications				Additional studies found in the reference list	Search in Google Scholar	Total number of studies included
	‘Urticaria keyword’	Relevant articles taken into consideration					
		Review articles	Case reports/letters	Original articles			
Systemic symptoms	136	0	0	0	0	0	0
Systemic involvement	48	0	0	0	0	1	1
Systemic complaints	2	0	0	1	0	2	2
Systemic manifestations	49	0	0	0	0	0	0
Organ involvement	22	0	0	0	0	0	0
Pulmonary system							
Pulmonary involvement	5	0	0	0	0	2	2
Asthma	105	0	1	1	1	0	1
Bronchial hyperreactivity	1	0	0	1	0	6	2
Bronchial hyperresponsiveness	2	0	0	2	0	5	2
Upper respiratory tract	1	0	1	0	0	0	1
Larynx	0	0	0	0	0	1	1
Pharynx	0	0	0	0	0	2	0
Nose	0	0	0	0	0	1	1
Eye	4	0	0	0	0	0	0
Neurological system							
Nervous system	2	0	0	0	0	2	2
Brain	91	0	0	1	0	0	1
Cerebellum	3	0	0	1	0	1	1
Central nervous system	0	0	0	0	0	0	0
Peripheral nervous system	0	0	0	0	0	0	0
Cardiovascular system							
Cardiac system	0	0	0	0	0	0	0
Cardiac symptoms	0	0	0	0	0	0	0
Cardiologic symptoms	0	0	0	0	0	3	2
Cardiac involvement	1	1	0	0	0	1	1
Heart	0	0	0	0	0	3	0
Kounis syndrome	11	0	5	0	2	4	5
Hypertension	85	0	0	2	0	2	2
Blood vessels	44	0	0	0	0	0	0
Veins	0	0	0	0	0	0	0
Arteries	0	0	0	0	0	5	3
Lymphedema	5	0	0	0	0	0	0
Lymphadenopathy	57	0	0	0	0	0	0
Haematological system	0	0	0	0	0	0	0
Gastrointestinal system							
Gastritis	60	0	0	1	0	2	3
Gastrointestinal symptoms	0	0	0	0	0	1	1
Intestines	5	0	0	0	0	3	2
Gut	35	0	0	1	0	2	1
Duodenum	10	0	0	2	0	2	1
Esophagus	0	0	0	0	0	0	0
Liver	238	0	2	0	0	2	2
Pancreas	9	0	0	3	0	1	1
Gallbladder	2	0	2	0	0	2	0
Genitourinary system							
Genitourinary system	0	0	0	0	0	1	1
Fertility	4	0	0	0	0	0	0
Musculoskeletal system							
Joints	33	0	0	0	0	2	2
Arthralgia	133	0	1	0	0	1	0
Skeletal system	5	0	0	0	0	0	0
Bones	20	0	1	0	1	0	2
Renal system							
Renal system	0	0	0	0	0	0	0
Kidneys	17	0	0	0	0	0	0
Renal involvement	10	0	0	0	0	0	0
Renal functions	1	0	0	0	0	0	0

**Table 2:** Supporting references by body system

Body system	Reference number	Type of report	Key content
Respiratory	[11]	Prospective study	Pulmonary function tests revealed that 22/26 of CSU patients had asthma or abnormal bronchial reactivity
	[12]	Prospective study	Bronchial hyperreactivity was present in 13/30 of cholinergic urticaria patients which was higher than CSU patients and healthy controls
	[13]	Prospective study	Dermographism patients exhibited increase in airway resistance and a decrease in specific airway conductance
	[10]	Case report	Authors reported two CU patients in whom urticaria onset was associated with the simultaneous appearance of asthmatic symptoms
Gastrointestinal system	[14]	Prospective study	Forty-four percent of 330 CU patients described abdominal problems mainly gastritis
	[16]	Prospective study	After duodenal histamine challenge, 5 of 7 CU patients showed systemic symptoms including diarrhoea and in 6 of 7 histopathological examination revealed ultrastructural changes in the duodenal tissue
	[17]	Prospective study	Mast cell numbers were significantly increased in the stomach and duodenum of CU patients even if they did not have GIS symptoms
	[21]	Prospective study	Gastroduodenal and intestinal permeability were significantly increased in patients with CU
	[22]	Case report	A patient with cholinergic urticaria and erythematous pangastritis, whose severe dyspeptic complaints did not resolve with proton pump inhibitors but did with omalizumab
	[23]	Case report	Transient hepatocellular injury including elevation of liver enzymes and hepatosplenomegaly during cholinergic urticaria attacks
Cardiovascular system	[39]	Prospective study	Hypertension is associated with extended duration of CIU
	[38]	Retrospective study	CIU patients had a 1.37-fold greater risk of developing hypertension than the non-CIU cohort
	[33–37]	Case report	The authors described Kounis syndrome resulting from acute urticaria, chronic urticaria, autoimmune urticaria and cold urticaria
Central nervous system	[42]	Prospective study	By using multiple-modality brain imaging tools, the authors demonstrated dysfunction of the striatum in CSU patients
	[43]	Prospective study	Authors demonstrated the altered cerebellar activity and cerebellum-reward-sensorimotor loops in CSU
	[40]	Case report	Authors described a patient with cholinergic urticaria associated with epileptic seizures and abnormalities on the encephalogram
	[41]	Case report	Authors reported a patient with an unusual form of migraine with urticarial lesions appearing at the end of each migraine attack
Musculoskeletal system	[47]	Retrospective study	1035 (8.7%) patients with CU were diagnosed with osteoporosis compared with 4046 (6.8%) controls. The adjusted multivariate analysis demonstrated that CU was significantly associated with a higher risk for osteoporosis (HR = 1.23, 95% CI 1.10–1.37, P < 0.001)
	[44]	Case series	Authors reported four cases with urticaria, angioedema, articular manifestations and HLA-B51 positivity
	[45]	Case series	Authors described 9 patients with angioedema, hives and arthritis which they referred as AHA syndrome
	[46]	Case report	Authors described a patient whose CU was associated with autoimmune thyroid disease and both CU and arthralgia responded to treatment with l-thyroxine

The references are ranked according to evidence quality

*CU* chronic urticaria, *CIU* chronic idiopathic urticaria, *CSU* chronic spontaneous urticaria

## Respiratory system (search terms employed urticaria and ‘upper respiratory tract’, ‘lower respiratory tract’, ‘pharynx’, ‘larynx’, ‘nose’, ‘respiratory symptoms’, ‘pulmonary system’, ‘asthma’, ‘bronchial hyperresponsiveness’)

Zuberbier et al. reported dyspnoea, rhinorrhoea or irritation of the eyes in 9.1% of urticaria patients [9]. Tedeschi et al. reported two autoimmune/autoreactive CU patients in whom urticaria onset was associated with the simultaneous appearance of asthmatic symptoms [10]. The same Italian group investigated pulmonary function and bronchial hyperresponsiveness in a group of patients with CU [11]. They included 26 CU patients and 26 asthmatic patients as controls and performed pulmonary function tests with methacholine provocation during a phase of moderate disease activity. 5 out of 26 had hypersensitivity to grass, HDM, olive, ragweed. The authors found that 22/26 (85%) of CSU patients had asthma or abnormal bronchial reactivity. Airway hyperresponsiveness was not associated with gender, disease duration, intolerance of NSAIDs, positive autologous serum skin testing or respiratory allergy. They concluded that results of the present study are completely different in that 85% of the patients with CU were found to have bronchial hyperresponsiveness (BHR) or, in some cases, overt asthma; this proportion largely exceeded the one expected in a normal population (5–20%). Petalas et al. studied cholinergic urticaria patients and found that bronchial hyperresponsiveness (BHR) was present in 13 of 30 (43%) of them but in only 1 of 15 patients with CU (7%), and 1 of 14 healthy volunteers (7%); the observed difference was statistically significant. In addition, a statistically significant correlation was found between patient age and disease duration and between intensity and frequency of symptoms [12]. Henz et al. found increased BHR in subjects with symptomatic dermographism and proposed a link between cutaneous and bronchial hyperreactivity, which might be based on a subclinical activation of mast cells that would induce changes in receptor expression and responsiveness of resident bronchial and cutaneous tissue and infiltrating inflammatory cells [13]. Even though clinical observations suggest CSU does not involve larynx, Juhlin et al. reported 8 from 330 CU patients to have laryngeal edema [14].

## Gastrointestinal system (search terms employed urticaria and ‘gastrointestinal system’, ‘gastrointestinal symptoms’, ‘esophagus’, ‘intestines’, ‘gastric’, ‘gut’, ‘colon’, ‘duodenum’ ‘liver’, ‘pancreas’, ‘gall bladder’)

Nausea, vomiting and epigastric abdominal pain are present in around 40% of CU patients; the term “Chronic Gastrointestinal Urticaria” has been used [14, 15]. Since the gut is frequently exposed to histamine from foods, the action of histamine on the digestive tract has been investigated. Kanny et al. examined duodenal biopsies from seven patients with CU before and after intraduodenal administration of histamine (120 mg). Five CU patients had clinical symptoms (diarrhoea, urticaria, headache, accelerated heart rate, and drop in blood pressure) within 1 h of duodenal histamine challenge (DHC). Ultrastructural changes, including oedema of the interstitial tissue, enlargement of the basal intercellular spaces, slight congestion of the endothelial cells and pericapillary oedema were observed in six patients 45 min after DHC. The authors concluded that histamine can induce oedema in the basal intercellular spaces of the duodenal mucosa and in the submucosa without evident change in the integrity of intercellular junctions [16]. Minnei et al. investigated if the mast cell number in the gastroduodenal mucosa is increased in CU patients compared to controls and found that CU patients demonstrated a significantly increased mast cell number in the stomach and the duodenum even if they did not have gastrointestinal symptoms [17]. Teramura et al. recently reported a patient with diarrhoea and urticaria in whom the symptoms were linked to intestinal oedema caused by urticaria itself [18].

Intestinal permeability studies were performed by Andre´ et al. who reported a slightly elevated lactulose/mannitol index in CU patients, whilst Guida et al. reported normal intestinal permeability in all patients with CU [6, 19, 20]. Buhner et al. designed a study to investigate whether disturbances of the gastrointestinal barrier function play a role in the pathomechanism of CU by enrolling 55 patients with CU. Gastrointestinal permeability was measured with an in vivo triple-sugar-test before and after 24 days of a low pseudoallergen diet. Sucrose served as marker of gastroduodenal permeability and lactulose/mannitol ratio for small intestinal permeability. They found that basal gastroduodenal and intestinal permeability were significantly increased in patients with urticaria by comparison with controls. In 29 of the 55 patients urticarial symptoms decreased or completely disappeared during the diet (responders). Compared to non-responders, responders had significantly higher gastroduodenal permeability before the diet, which decreased after the diet (0.17 ± 0.02; P < 0.001). The results of the study suggested that an impaired gastroduodenal barrier function may be of pathophysiological importance in the development of pseudoallergy in CU patients [21].

A case report from Turkey described a patient with cholinergic urticaria and erythematous pangastritis whose severe dyspeptic complaints did not resolve with proton pump inhibitors but with omalizumab [22].

### The liver

Searching for “urticaria and liver” revealed a case of cholinergic urticaria with transient hepatocellular injury during attacks of cholinergic urticaria in a 25-year-old man with ulcerative colitis [23]. We could not find any other publications relating to liver dysfunction in CU but we found reports of “yellow urticaria” with high blood bilirubin levels due to various liver diseases. The unusual colour of the lesions was thought to be due to increased permeability of cutaneous blood vessels in urticaria resulted in exudation of bilirubin into the weals [24]. There were no reports of gall bladder dysfunction.

### The pancreas

Barba et al. investigated the exocrine function of the pancreas in 25 patients with CU by analysing the quantitative or qualitative deficiency of pancreatic enzyme secretion. All the patients showed normal faecal chymotrypsin excretion and 23/25 had normal bentiromide (para-amino benzoic acid) and pancreolauryl tests [25]. The authors concluded that these findings did not support pancreatic deficiency in CU.

## Cardiovascular system (search terms employed urticaria and ‘cardiac involvement’ ‘heart’, ‘cardiologic symptoms’ ‘hypertension’)

Histamine is found in high concentrations in the healthy heart and in very high concentrations in the coronary arteries of patients known to have died from coronary heart disease [26, 27]. Ginsburg et al. Demonstrated histamine to be capable of inducing coronary artery spasm [28]. In vitro studies of the human heart have revealed that the H1 receptors mediate contraction of coronary vascular smooth muscle, while H2 receptors mediate relaxation [29]. During hypersensitivity reactions or anaphylaxis, degranulation of cardiac mast cells will lead to release of vasoactive substances with reduction in coronary blood flow and depression in regional myocardial contractile function [30].

The concurrence of acute coronary syndromes with conditions associated with mast cell degranulation has been termed Kounis syndrome (KS) after his description in 1991 [31]. Three types of KS has been described: type I variant (most common variant, 72.6%) is characterized by the release of inflammatory mediators which induces coronary artery spasm with or without increase of cardiac enzymes and troponins. Type II variant (22.3%) is featured with the release of inflammatory mediators which induces coronary artery spasm together with plaque erosion or rupture manifesting as acute MI. Type III variant (5.1%) includes patients with coronary artery stent thrombosis as a result of an allergic reaction [32]. Allergic, anaphylactic and anaphylactoid reactions are associated with this syndrome [32]. Recently, Erxun et al. described a 31 year old patient with Kounis syndrome who had been suffering from chronic autoimmune urticaria for 3 years. Her urticaria became more serious 1 week before an emergency department (ED) visit with heart palpitations, precordial pain, chest tightness and excessive sweating. Prior to this acute presentation, her heart symptoms had tended to resolve about 2 h after the urticaria disappeared. She had repeated attacks of urticaria exacerbations with cardiac symptoms at follow up. An ECG showed ST-segment elevation and, on review of her ED visits, the ST-segment elevation was found to occur almost at the same time she was suffering from serious urticaria, but it returned to normal when her urticaria disappeared. Angiography was performed during and after heart symptoms; the results revealed a coronary artery spasm at the time of onset of her heart symptoms that returned to normal when her symptoms were relieved. The authors stressed that when severe chronic urticaria is accompanied by repeated heart symptoms, physicians should consider the possibility of Kounis syndrome [33]. A case of cold-induced urticaria complicated by Kounis syndrome during swimming in seawater was also reported who had a normal ECG but a high cardiac troponin [34]. Kounis syndrome has more commonly been reported in acute urticaria and anaphylaxis but it could also be more common in acute severe chronic urticaria outbreaks than is currently recognised [35–37].

Chang et al. reported that hypertension was associated with CIU. Their patient cohort had a 1.37-fold greater risk of developing subsequent hypertension than controls. They attributed this association to alterations in the blood coagulation and fibrinolysis pathways as well as systemic inflammation associated with CIU [38]. In another study, Nebiolo et al. reported that systemic hypertension was present in 42 of 228 patients (18.9%) and that hypertension is associated with extended duration of CIU [39].

## Central nervous system (search terms employed urticaria and ‘brain’, ‘neurologic symptoms’, ‘central nervous system’)

The search for “urticaria and brain” revealed a case of cholinergic urticaria with seizures. Harada et al. reported a 10-year-old boy with cholinergic urticaria associated with epileptic seizures and abnormalities on electroencephalogram. They proposed that sweat-promoting stimuli, such as heat, exercise and tension stimulate the autonomic centre in the diencephalon or brain stem, and that excitation of the autonomic centre is transmitted to the efferent sympathetic nerves, causing cholinergic urticaria. When the intensity of stimulation is high, the autonomic centre exhibits abnormal activities and causes epileptic seizures [40]. Fumal et al. reported a patient with an unusual form of migraine with urticarial lesions on the chest at the end of each migraine attack outlasting the attack by 10 to 90 min, which was assumed to be related to a systemic release of serotonin or other vasoactive substances like histamine, bradykinin, or nitric oxide [41].

Wang et al. investigated the functional and structural alterations of the striatum in chronic spontaneous urticaria (CSU). They performed amplitude of low frequency fluctuations (ALFF), voxel-based morphometry (VBM), and seed-based resting-state functional connectivity (rs-FC) analysis on 40 CSU patients and 40 healthy controls to assess brain activity and related plasticity. They found that CSU patients had higher ALFF values in the right ventral striatum/putamen, which were positively associated with clinical symptoms as measured by UAS7; a higher gray matter volume in the right ventral striatum and putamen; and decreased rs-FC between the right ventral striatum and the right occipital cortex and between the right putamen and the left precentral gyrus. In summary, by using multiple-modality brain imaging tools, they demonstrated dysfunction of the striatum in CSU. The authors concluded that CSU may be associated with disrupted reward, motivation, and motor processing [42]. These investigators published a recent original article which demonstrated the altered cerebellar activity and cerebellum-reward-sensorimotor loops in CSU; these changes in cerebellar activity were shown to decline with treatment when urticarial activity is decreased [43].

## Musculoskeletal system (search terms employed urticaria and ‘skeletal system’, ‘joints’)

A paper from 1989 reported four HLA-B51 positive patients with simultaneous urticarial and articular manifestations but without evidence of immune complex disease. The authors suggested that a common genetic background might be present in some cases of urticaria with articular complaints [44]. McNeil et al. described 9 patients with concurrent arthritis and/or arthralgia, urticaria and angioedema in whom routine laboratory studies were normal, including complement levels, humoral and cellular immunity. The absence of associated infection and connective tissue disease suggested this recurrent triad represents a distinct entity, which the authors designated the AHA (arthritis, hives and angioedema) syndrome [45]. A case report from 2007 described a patient with CU and mild arthritis associated with autoimmune thyroid disease who was successfully treated with l-thyroxine. Both urticaria and arthralgia responded to treatment [46].

Recently, an association between CU and osteoporosis was reported [47]. In this report, CU was associated with lower serum PTH, lower rates of hypocalcemia and higher rates of osteoporosis. Patients with osteoporosis and CU were younger more likely to be of male sex and had a higher prevalence of obesity than osteoporotic patients without CU, further strengthening the association between CU and osteoporosis.

## Renal system (search terms employed urticaria and ‘renal involvement’, ‘kidneys’, ‘renal functions’) and genitourinary system (‘genitourinary system’ and ‘fertility’)

We found no reports of nephritis, proteinuria or haematuria associated with chronic urticarial activity. However, interference with micturition due to delayed pressure urticaria swelling after sexual intercourse has been reported [48].

## Conclusions

Systemic consequences of urticaria could result from increased circulation of cutaneous mast cell mediators, generalized enhancement of releasability of mast cells, a reactive increase in mast cell numbers (as opposed to clonal in mastocytosis), increase in plasma histamine level or all of these. It might be expected that any systemic effect would be most obvious when the disease is most active. The natural history of CU is one of natural improvement followed by resolution so looking for evidence of dysfunction would be logical during the acute stage of the illness or during acute urticaria. Patients presenting acutely unwell with severe urticaria are often treated with short courses of systemic steroids that are likely to mask subclinical effects of the illness.

**Fig. 1:**
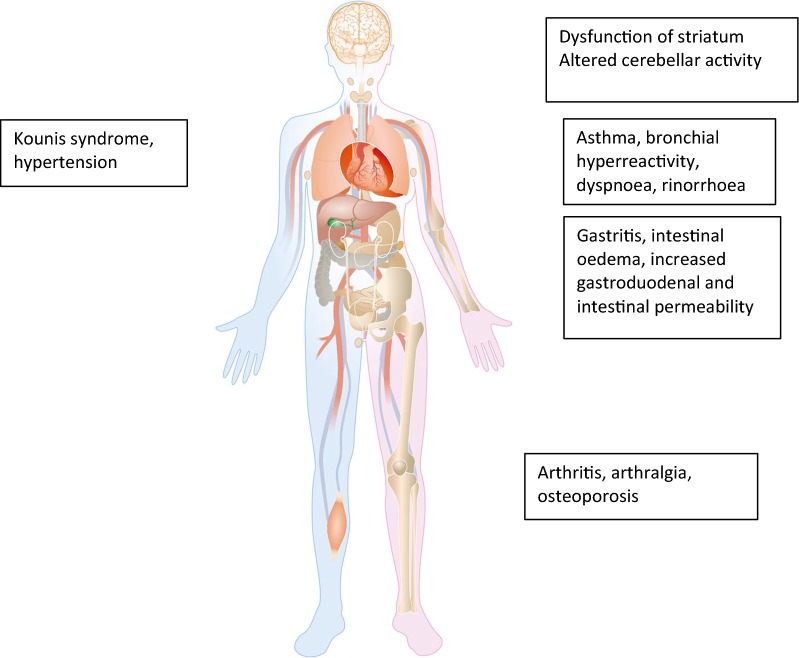
Involved systems in chronic urticaria and relevant manifestations

Searching for reports of organ-based dysfunction in patients with urticaria revealed some evidence in cardiac, respiratory, gastrointestinal, central nervous and musculo-skeletal systems (Fig. 1). The relative paucity of published reports does not exclude organ-based dysfunction if a relationship has not been looked for or written up in the medical literature. The relevance of these findings needs to be further determined. However, they justify prospective studies in larger numbers of patients and at different stages of disease activity.

Limitations of this review include:

1. The possibility that relevant reports were missed in the literature search by using incomplete search terms.
2. The anecdotal reports may have been co-incidental rather than directly related to urticaria.
3. It was not possible to assess the effects of urticaria treatment on potential improvement of any organ-based dysfunction without it.

## Data Availability

Data sharing not applicable to this article as no datasets were generated or analysed during the current study.

## Abbreviations

ALFF: amplitude of low frequency fluctuations
BHR: bronchial hyperresponsiveness
CU: chronic urticaria
CSU: chronic spontaneous urticaria
CIU: chronic idiopathic urticaria
DHC: duodenal histamine challenge
ED: emergency department
Rs-FC: seed-based resting-state functional connectivity
VBM: voxel-based morphometry

## References

[1] Zuberbier T, Aberer W, Asero R, Abdul Latiff AH, Baker D, Ballmer-Weber B (2018). The EAACI/GA^2^LEN/EDF/WAO guideline for the definition, classification, diagnosis and management of Urticaria. The 2017 revision and update. Allergy.

[2] Grattan C, Maurer M, Zuberbier T, Grattan C (2010). Aetiopathogenesis of urticaria. Urticaria and angioedema.

[3] Füreder W, Agis H, Willheim M, Bankl HC, Maier U, Kishi K (1995). Differential expression of complement receptors on human basophils and mast cells. Evidence for mast cell heterogeneity and CD88/C5aR expression on skin mast cells. J Immunol..

[4] Ferrer M, Nazakawa K, Kaplan AP (1999). Complement-dependence of histamine release in chronic urticaria. J Allergy Clin Immunol.

[5] Ferrer M, Nuñez-Córdoba JM, Luquin E, Grattan CE, De la Borbolla JM, Sanz ML (2010). Serum total tryptase levels are increased in patients with active chronic urticaria. Clin Exp Allergy.

[6] Guida B, De Martino CD, De Martino SD, Tritto G, Patella V, Trio R (2000). Histamine plasma levels and elimination diet in chronic idiopathic urticaria. Eur J Clin Nutr.

[7] Son JH, Chung BY, Kim HO, Park CW (2018). A histamine-free diet is helpful for treatment of adult patients with chronic spontaneous urticaria. Ann Dermatol.

[8] Doong JC, Chichester K, Oliver ET, Schwartz LB, Saini SS (2017). Chronic idiopathic urticaria: systemic complaints and their relationship with disease and immune measures. J Allergy Clin Immunol Pract..

[9] Zuberbier T, Balke M, Worm M, Edenharter G, Maurer M (2010). Epidemiology of urticaria: a representative cross-sectional population survey. Clin Exp Dermatol.

[10] Tedeschi A, Contini M, Asero R (2009). Simultaneous occurrence of chronic autoimmune urticaria and non-allergic asthma: a common mechanism?. Eur Ann Allergy Clin Immunol.

[11] Asero R, Madonini E (2006). Bronchial hyperresponsiveness is a common feature in patients with chronic urticaria. J Investig Allergol Clin Immunol.

[12] Petalas K, Kontou-Fili K, Gratziou C (2009). Bronchial hyperresponsiveness in patients with cholinergic urticaria. Ann Allergy Asthma Immunol.

[13] Henz BM, Jeep S, Ziegert FS, Niemann J, Kunkel G (1996). Dermal and bronchial hyperreactivity in urticarial dermographism and urticaria factitia. Allergy.

[14] Juhlin L (1981). Recurrent urticaria: clinical investigation of 330 patients. Br J Dermatol.

[15] Kalas D, Pronai L, Ferenczi K, Palos G (1996). Connection between *Helicobacter pylori* infection and chronic gastrointestinal urticaria. Orv Hetil.

[16] Kanny G, Grignon G, Dauca M, Guedenet JC, Moneret-Vautrin DA (1996). Ultrastructural changes in the duodenal mucosa induced by ingested histamine in patients with chronic urticaria. Allergy.

[17] Minnei F, Wetzels C, De Hertogh G, Van Eyken P, Ectors N, Ambu R (2006). Chronic urticaria is associated with mast cell infiltration in the gastroduodenal mucosa. Virchows Arch.

[18] Teramura K, Minami S, Yamaguchi A, Tanaka T, Fujimoto N (2017). Infectious urticaria complicated with intestinal edema. J Dermatol.

[19] Andre´ C, Andre´ F, Colin L (1989). Effect of allergen ingestion challenge with and without cromoglycate cover on intestinal permeability in atopic dermatitis, urticaria and other symptoms of food allergy. Allergy.

[20] Sequeira IR, Lentle RG, Kruger MC, Hurst RD (2014). Standardising the lactulose mannitol test of gut permeability to minimise error and promote comparability. PLoS ONE.

[21] Buhner S, Reese I, Kuehl F, Lochs H, Zuberbier T (2004). Pseudoallergic reactions in chronic urticaria are associated with altered gastroduodenal permeability. Allergy.

[22] Kutlu A, Tanoglu A, Ozturk S (2015). Healing effects of omalizumab in a patient with cholinergic urticaria associated severe dyspeptic complaints. Chin Med J (Engl)..

[23] Niv Y, Elkan I, Fraser GM (1997). Transient hepatocellular injury during attacks of cholinergic urticaria. Isr J Med Sci.

[24] Combalia A, Fustà X, Guilabert A, Mascaró JM, Estrach T (2017). Yellow urticaria: report of two cases and review of the literature. J Eur Acad Dermatol Venereol.

[25] Barba A, Schena D, Chieregato GC, Riela A, Brocco G, Scuro LA, Cavallini G (1988). Exocrine pancreatic function in chronic urticaria patients is normal. Dermatologica..

[26] Mannaioni PF (1972). Physiology and pharmacology of cardiac histamine. Arch Int Pharmacodyn Ther.

[27] Kalsner S, Richards R (1984). Coronary arteries of cardiac patients are hyperreactive and contain stores of amines: a mechanism for coronary spasm. Science.

[28] Ginsburg R, Bristow MR, Kantrowitz N, Baim DS, Harrison DC (1981). Histamine provocation of clinical coronary artery spasm: implications concerning pathogenesis of variant angina pectoris. Am Heart J.

[29] Ginsberg R, Bristow MR, Stinson EB, Harrison DC (1980). Histamine receptors in the human heart. Life Sci.

[30] Ito BR, Engler RI, del Balzo U (1993). Role of cardiac mast cells in complement C5a induced myocardial ischemia. Am J Physiol.

[31] Kounis NG, Zavras GM (1991). Histamine-induced coronary artery spasm: the concept of allergic angina. Br J Clin Pract.

[32] Kounis NG (2016). Kounis syndrome: an update on epidemiology, pathogenesis, diagnosis and therapeutic management. Clin Chem Lab Med.

[33] Erxun K, Wei L, Shuying Q (2016). Kounis syndrome caused by chronic autoimmune urticaria: a case report. J Emerg Med.

[34] Mazarakis A, Bardousis K, Almpanis G, Mazaraki I, Markou S, Kounis NG (2014). Kounis syndrome following cold urticaria: the swimmer’s death. Int J Cardiol.

[35] Vaswani SK, Plack RH, Norman PS (1996). Acute severe urticaria and angioedema leading to myocardial infarction. Ann Allergy Asthma Immunol.

[36] Gupta MK, Gupta P, Rezai F (2001). Histamine—can it cause an acute coronary event?. Clin Cardiol.

[37] Connor S, Child N, Burdon-Jones D, Connor A (2010). Cardiac arrest secondary to type 2 Kounis syndrome resulting from urticaria and angioedema. Emerg Med J..

[38] Chang HW, Cheng HM, Yen HR, Hsu CY, Lee YC, Chiang JH (2016). Association between chronic idiopathic urticaria and hypertension: a population-based retrospective cohort study. Ann Allergy Asthma Immunol.

[39] Nebiolo F, Bergia R, Bommarito L, Bugiani M, Heffler E, Carosso A (2009). Effect of arterial hypertension on chronic urticaria duration. Ann Allergy Asthma Immunol.

[40] Harada T, Yamamura Y, Ishizaki F, Hide M, Morita E, Inoue K (2001). A case of cholinergic urticaria with epileptic seizure and abnormalities on electroencephalogram. No To Shinkei..

[41] Fumal A, Crémers J, Ambrosini A, Grand JL, Schoenen J (2006). Migraine with urticaria. Neurology..

[42] Wang Y, Fang JL, Cui B, Liu J, Song P, Lang C (2018). The functional and structural alterations of the striatum in chronic spontaneous urticaria. Sci Rep..

[43] Wang Y, Fang J, Sang P (2018). the dysfunction of the cerebellum and its cerebellum-reward-sensorimotor loops in chronic spontaneous urticaria. Cerebellum.

[44] Pasero G, Olivieri I, Gemignani G, Vitali C (1989). Urticaria/arthritis syndrome: report of four B51 positive patients. Ann Rheum Dis.

[45] McNeil DJ, Kinsella TD, Crawford AM, Fritzler MJ (1987). The AHA syndrome: arthritis, hives and angioedema. Rheumatol Int.

[46] Milchert M, Flicinski J, Ostenek L, Brzoska M (2007). Chronic urticaria and mild arthritis associated with autoimmune thyroid disease: successful treatment with L-thyroxine. Acta Derm Venereol.

[47] Shalom G, Kridin K, Babaev M, Magen E, Tiosano S, Dreiher J, Horev A, Khury R, Comaneshter D, Agmon-Levin N, Cohen AD (2019). Chronic urticaria and osteoporosis: a longitudinal, community-based cohort study of 11,944 patients. Br J Dermatol.

[48] Poon E, Kodza-Black A (1998). Delayed pressure urticaria causing obstruction of urinary flow. Acta Derm Venereol.

